# Divergent Enteroviruses from Macaques with Chronic Diarrhea

**DOI:** 10.1128/MRA.00699-21

**Published:** 2021-08-05

**Authors:** Kathie A. Mihindukulasuriya, Lindsay Droit, Margaret H. Gilbert, Peter J. Didier, Anne Paredes, Scott A. Handley, Rudolf P. Bohm, David Wang

**Affiliations:** a Department of Pathology, Washington University School of Medicine, St. Louis, Missouri, USA; b Department of Immunology, Washington University School of Medicine, St. Louis, Missouri, USA; c Tulane University Office of Research, Institutional Animal Care and Use Committee, Covington, Louisiana, USA; d Division of Veterinary Medicine, Tulane National Primate Research Center, Covington, Louisiana, USA; e Division of Comparative Pathology, Tulane National Primate Research Center, Covington, Louisiana, USA; Queens College CUNY

## Abstract

We report the draft genome sequences of five novel members of the family *Picornaviridae* that were isolated from the stool of rhesus macaques (Macaca mulatta) with chronic diarrhea. The strains were named NOLA-1 through NOLA-5 because the macaques were residents of the Tulane National Primate Research Center.

## ANNOUNCEMENT

Rhesus macaques are the most widely used primates in medical research and have been critical for research on HIV, Zika virus, and tuberculosis. Rhesus macaques and other nonhuman primates have been important for translational research because they are the closest animal models to humans, genetically, physiologically, and behaviorally (1). Viral infections of rhesus macaques can affect their health and also affect the results of experimental studies.

In a previous study, the viromes of 15 rhesus macaques that had been hospitalized with intractable diarrhea for at least 3 months were analyzed (2). The genomes of NOLA-1 through NOLA-5 were detected in stool samples that had been sequenced following enrichment for RNA and DNA viruses as described previously (2). Briefly, approximately 100 to 200 mg of frozen stool was resuspended in buffer and filtered through 0.45-μm filters until clarified. Clarified samples were subsequently treated with lysozyme to liberate bacterial nucleic acid, followed by DNase treatment to remove nonencapsidated nucleic acid. Total nucleic acid (both RNA and DNA) was extracted on a COBAS AmpliPrep instrument (Roche), using the AmpliPrep total nucleic acid isolation kit, according to the manufacturer’s recommendations. Purified total nucleic acid was reverse transcribed, and the second strand was synthesized, PCR amplified using barcoded primers consisting of a base-balanced 16-nucleotide specific sequence, and used for NEBNext library construction (New England BioLabs). Libraries were multiplexed (12 samples per flow cell) on an Illumina MiSeq instrument (Washington University Center for Genome Sciences) using a paired-end 2 × 250-bp protocol. Potential viral sequences were identified with VirusSeeker-Virome v0.063 and assembled with VirusSeeker-Discovery v0.063 (3). Four of the genomes (NOLA-2, NOLA-3, NOLA-4, and NOLA-5) were finished using the 3′ RACE system for rapid amplification of cDNA ends kit (Thermo Fisher Scientific).

Genomes were annotated and prepared for submission using VAPiD v1.6.6 (4), followed by manual curation of the resulting GenBank files, including reassembly of the raw reads by MEGAHIT v1.1.4 (5, 6) followed by metaFlye v2.7.1 (7, 8) as needed; the files were then reformatted for submission via gbf2tbl.pl and tbl2asn, following instructions provided (https://github.com/jbadomics/genbank_submit/blob/master/README.md). This study was approved by the Institutional Animal Care and Use Committee of Tulane University National Primate Research Center (permission number IACUC R24OD019793).

The putative polyproteins encoded by these genomes are similar to those of other enteroviruses. Enterovirus J strain NOLA-1 and enterovirus J strain NOLA-3 are 85% and 82% identical, respectively, at the nucleotide level to simian picornavirus enterovirus J strain POo-1 (GenBank accession number FJ007373.1). Enterovirus J strain NOLA-1 is 7,316 bp in length with a GC content of 45%, while enterovirus J strain NOLA-3 is 7,243 bp in length with a GC content of 45%. Simian enterovirus SV19 strain NOLA-2 is 83% identical to simian enterovirus SV19 strain M19s (GenBank accession number AF326754.2). Simian enterovirus SV19 strain NOLA-2 has a length of 7,232 bp with a GC content of 44%. Simian enterovirus SV19 strain NOLA-4 and simian enterovirus SV19 strain NOLA-5 are 83% and 84%, respectively, identical to simian enterovirus SV19 isolate cg4006 (GenBank accession number KT961654.1). Simian enterovirus SV19 strain NOLA-4 is 6,907 bp in length with a GC content of 45%, while simian enterovirus SV19 strain NOLA-5 is 6,400 bp in length with a GC content of 46%.

### Data availability.

The draft genome sequences reported here were deposited in DDBJ/ENA/GenBank under the accession numbers MZ312496, MZ312495, MZ312494, MZ312493, and MZ312497. Raw data were deposited in the Sequence Read Archive (SRA) (accession number PRJNA491509).
